# Research('s) Sweet Hearts: Experimental Biomedical Models of Diabetic Cardiomyopathy

**DOI:** 10.3389/fcvm.2021.703355

**Published:** 2021-07-23

**Authors:** Claudia Richter, Rabea Hinkel

**Affiliations:** ^1^Laboratory Animal Science Unit, German Primate Center, Leibniz Institute for Primate Research, Goettingen, Germany; ^2^Partnersite Goettingen, German Center for Cardiovascular Research (DZHK e.V.), Goettingen, Germany; ^3^Stiftung Tierärztliche Hochschule Hannover, University of Veterinary Medicine, Hanover, Germany

**Keywords:** diabetes, cardiovascular, cardiomyopathy, animal model, arrhythmia

## Abstract

Diabetes and the often accompanying cardiovascular diseases including cardiomyopathy represent a complex disease, that is reluctant to reveal the molecular mechanisms and underlying cellular responses. Current research projects on diabetic cardiomyopathy are predominantly based on animal models, in which there are not only obvious advantages, such as genetics that can be traced over generations and the directly measurable influence of dietary types, but also not despisable disadvantages. Thus, many studies are built up on transgenic rodent models, which are partly comparable to symptoms in humans due to their genetic alterations, but on the other hand are also under discussion regarding their clinical relevance in the translation of biomedical therapeutic approaches. Furthermore, a focus on transgenic rodent models ignores spontaneously occurring diabetes in larger mammals (such as dogs or pigs), which represent with their anatomical similarity to humans regarding their cardiovascular situation appealing models for testing translational approaches. With this in mind, we aim to shed light on the currently most popular animal models for diabetic cardiomyopathy and, by weighing the advantages and disadvantages, provide decision support for future animal experimental work in the field, hence advancing the biomedical translation of promising approaches into clinical application.

## 1. Introduction

In the last decades, diabetes mellitus has advanced to become a common disease, with four main types being distinguished since 1998. Thus, patients are divided into either type 1 diabetes mellitus (T1DM), characterized by an absolute insulin deficiency due to autoimmune regulated destruction of pancreatic β-cells, and type 2 diabetes mellitus (T2DM), which is due to different combinations of insulin resistance, hyperinsulinism, relative insulin deficiency and secretory disorders. In addition, there are genetic diabetes or diabetes triggered by other disease syndromes and toxins, respectively, which is divided into different subtypes depending on the trigger, as well as gestational diabetes (1–3).

Along with diabetes, the risk of cardiovascular disease increases rapidly. In fact, hyperglycaemic mortality in diabetic patients is closely related to macrovascular and conductional changes in the heart. Thus, diabetes represents one of the most rapidly developing and most important risk factors for cardiac arrhythmogenesis and vascular remodeling (4–10). Statistically, diabetic patients have a 40 % higher risk of atrial fibrillation (AF) than non-diabetic patients (11). In this context, the term diabetic cardiomyopathy (DC) was established and stands in general for myocardial changes in function and tissue structure associated with diabetes as basic disease, but no direct attribution to other diseases such as coronary artery disease (CAD) (12–14). In fact, the appearance of DC is very complex and involves a wide variety of factors. These factors include, on the one hand, diabetic effects on the cardiomyocytes themselves, which show oxidative stress and hypertrophic signs in addition to metabolic changes. Second, this factors include vascular effects. These can be endothelial as well as microvascular dysfunction or artherosclerosis. All of these symptoms play into the clinical picture of diabetic cardiomyopathy, which in turn amounts to the risk for ischemia and arrhythmia due to the increased incidence for inflammation, fibrosis, and autonomic dysfunction (15–17).

One reason for the rapid spread of DC is the fact that T2DM in particular often occurs together with obesity. Obesity itself is an epidemic problem that affects all segments of the population and brings with it other health problems besides diabetes, such as cardiovascular changes, respiratory failure, muscle weakness and cancer (18). Furthermore, obesity often sets in motion a spiral of pre-diabetic symptoms that progress from metabolic syndrome associated with hypertension and often creeping development of insulin resistance to serious cardiac disease. This also comes into effect with regard to cardiac arrhythmia, such as atrial fribrillation (9). In addition, diabetic-triggered inflammation and deregulated oxidative energy metabolism of cardiomyocytes also cause micro- and macrovascular changes in patients, with overproduction of reactive oxygen species (ROS) and mitochondrial dysfunction in particular increasingly coming into research focus (19–22).

Taking all the above mentioned facts and the cumulative effect of diabetes mellitus and cardiovascular disease patterns, an extremely complex picture of DC emerges with many questions still unanswered. To address the complexity of diabetic cardiomyopathy and its physiological aftermath, it is essential to better understand the underlying molecular mechanisms. Finding and using the right animal models to investigate these relationships is hence one of the top priorities. Admittedly, a lot has already been published in the field of diabetic animal models [see e.g., (23, 24)]. However, especially with regard to cardiovascular and electrophysiological conditions, caution is required when choosing the right study object. The aim of this concise review is not to demonize individual established animal models, but to help in the choice of the right basic or translational biomedical model. Therefore, in the following sections the most common diabetic lab animal models are compared to cardiac animal models and the translational suitability in the preclinical phase is shown.

## 2. Biomedical Modeling of Diabetes and Cardiovascular Diseases

In principle, the actual research question should precede every experiment. Generally speaking this means that one should first be clear about which clinical symptomatology is to be represented and investigated in the animal model. This also determines which animal model will be the most suitable in the end. Besides, the question of clinical relevance plays an important role, since it is one thing to search for the basic regulatory mechanisms or genetic channel myopathies and another to establish completely new treatment strategies for human therapy. As a brief overview the below described diabetic approaches are summarized in Table 1.

**Table 1:** Brief overview of the commonly applied approaches for diabetes induction.

**Diabetic approach**	**Animal**	**Type**	**Cardiac and systemic effects**	**Reference/s**
*Chemical Application* (either 1x high dose or several low dose protocols exist)				
•STZ – Dosage [mg/kg]: Rodents 35–200 Pigs/NHP 50–150 – Hyperglycemia 2 d post injection •Alloxan – Dosage [mg/kg]: Rodents 40–200 Large animals 50–75 – Hyperglycemia 5 d post injection	Mouse, Rat, Zebrafish, Pig, NHP	T1DM	diastolic & systolic function ↓ Hypertrophy ↑ Fibrosis ↑ Apoptosis ↑ mitochondrial function ↓ oxidative stress ↑ cardiac size ↓	e.g., (25–27)
*Diet-induced Obesity*				
•High-fat diet – up to 60 cal% of fat and low as 20 cal% of carbohydrates (Rodents) – also overfed schemes with up to 6x (fish) or 1.5-2x (large animals) daily feeding •for NHP also high-fructose and high-sucrose diet	Mouse, Rat, Zebrafish, Drosophila, Pig, NHP	T2DM	diastolic & systolic function ↓ Hypertrophy ↑ Fibrosis ↑ Artherosclerosis ↑ Glucose ↑ Insulin ↑↑ Cholesterol ↑↑ HDL ↑ metabolic syndrome ↑	e.g., (28–33)
*Surgical Approach*				
•(Partial) Removal of pancreatic cells – also in combination with STZ (up to 200 mg/kg) or Alloxan	Mouse, Rat, Pig, NHP, Rabbit, Dog	T1DM	Hyperglycemia ↑ plasma insulin = insulin resistance ↑	e.g., (26)
*Genetic Lines*				
OVE26 Akita BB NOD *ob/ob (Lep^*ob*^)* *db/db (Lepr^*db*^)* ZDF	Mouse, Rat, Zebrafish	T1DM T2DM	cardiac dysfunction ↑ mitochondrial dysfunction ↑ Fibrosis ↑ oxidative stress ↑ Apoptosis ↑ cardiac size ↑ Hypertrophy ↑ Glucose oxidation ↓ Lipotoxicity ↑	e.g., (25, 27, 34)
LDLR^−/−^ PCSK9 GIPR^*dn*^	Pig	T2DM	cardiac dysfunction ↑ Hypercholesterolemia (esp. LDL) ↑↑ HDL ↓ Fibrosis ↑ Artherosclerosis ↑	e.g., (35)

### 2.1. Small Animal Models

Choosing individual animal models, we have to consider the experimental methods used to induce diabetes. In addition to genetically modified models there are also other experimental approaches. Nevertheless, also small animal models have their cardiovascular importance (36, 37).

#### 2.1.1. Spontaneous Diabetic Models

As an example, there is of course the spontaneously occurring diabetes, which has so far been described mainly in large animals such as dogs, cats and primates. In addition, there are also the small animal models. Spontaneously occurring cases are mainly characterized by hyperglycemia and transient hyperinsulinemia, and the majority of the animals are obese (38).

Exemplary for this diabetic type is the BioBred (BB) rat based on a spontaneous mutation in the Wistar rat, which is characterized by an acute syndrome of hyperglycemia, hyperinsulinemia and ketoacidosis (39–41). In the BB rat, spontaneous diabetes occurs in approximately 30 % of the middle-aged stock (between 60 and 120 days of age) and, because of its stable weight percentage, it is one of the lean diabetic models. Intense pancreatic insulitis, which is attributed to immune-driven pathogenesis, continues to be conspicuous (42, 43). As a second lean rat model for spontaneously occurring diabetes, the Goto-Kazaki (GK) rat can also be mentioned. This is a rat line characterized by glucose intolerance and is predominantly used as a model for non-obese T2DM (44, 45). Here again, the Wistar rat serves as the strain background. The third in the group is the Wistar Bonn/Kobori (WBN/Kob) rat, which shows symptoms such as hyperglycemia, glucosuria, hyperinsulnemia and glucose intolerance only in males from 8 to 9 months of age. Furthermore, these animals show bradycardia and dysfunctions in the para- and sympathetic nervous system (46). Last but not least, the Spontaneous Diabetic Torii (SDT) rats are so-called non-obese T2DM models, which can develop hyperglycemia at the age of 20 weeks. These animals also survive for longer periods without specific insulin treatment. Furthermore, SDT rats show neovascularization without prior ischemia, which, however, requires further studies (47).

As a kind of standard model for the pathophysiology of autoimmune diseases, such as T1DM, the Nonobese Diabetic (NOD) mouse counts. In this mouse line, developed in Japan in 1974, infiltration of the pancreatic islets with innate immune cells occurs as early as the third or fourth week of age, causing insulitis. This mouse line requires constant insulin therapy to keep the animals alive. Also, the AKITA mouse is also one of the spontaneously occurring models. Due to a mutation of the insulin 2 gene in combination with the prevention of proinsulin, also in this line a severe T1DM develops at the age of 3–4 weeks. Without treatment, the animals rarely reach an age beyond 12 weeks (41, 46, 48).

#### 2.1.2. Experimentally Induced Models

Experimentally induced models for diabetic cardiovascular disease are very well documented by the Animal Models of Diabetic Complications Consortium (AMDCC). Guidelines for validation of these models can also be found here. According to this, a valid animal model for diabetic vascular disease should have at least one of the following characteristics: (1) insulin resistance, (2) dysmetabolic syndrome, (3) impaired glucose tolerance, (4) T2DM, or (5) T1DM associated with atherosclerosis, peripheral vascular disease, or microvascular disease (34).

Many species develop diabetes and its associated cardiovascular disease solely on the basis of a dietary switch to the so-called Western Diet (Diet-induced Obesity, DIO). This involves feeding high-fat and high-protein diets, which promotes obesity and subsequently triggers insulin resistance. Long-term obesity is not only a parameter for diabetic development of the organism, but also for cardiomyopathy and the associated favors of arrhythmias and ischemias (49). However, it should be mentioned that mouse lines in particular have an efficient lipoprotein clearance system and are thus less susceptible to DIO approaches alone (34). Here, genetic and DIO approaches are often combined. Although, there are already studies published showing high-fat diet induced cardiovascular alterations in a mouse model providing long-term observations (28). DIO can be used for most laboratory animals.

Pharmacological or chemical approaches can also be chosen as another induction protocol (50). Foremost among these are streptozotocin (STZ) and alloxan. STZ is a synthetic nitrosoureido glucopyranose derivative that was actually developed as an antibiotic in anti-tumor therapy and targets DNA degeneration (51). The underlying trigger for the diabetic symptoms is the destruction of islet cells. However, experiments with rats have also shown disadvantages of this method, because STZ also has a high potential for oncogenic activity. In terms of cardiovascular phenomena, STZ hearts show an effect on calcium (Ca^2+^) handling within the cardiomyocyte as well as a reduced activity of the sarcoplasmic reticulum Ca^2+^-ATPase (SERCA2), a reduced Ca^2+^-uptake and release, and an alteration of the mitochondrial Ca^2+^-cycle (52). STZ may also have an effect on cardiac contractile function by interfering with the p38 MAP kinase-dependent oxidative stress mechanism (53). But, the use of STZ to induce diabetic symptoms is also a double-edged sword. On the one hand, the most common application is the single high-dose protocol as well as the multiple low-dose protocol. When a high concentration of STZ is administered once (e.g., 180 mg/kg in mice), diabetes develops in all animals within a few days, but mortality increases significantly at the same time. In contrast, this is not so pronounced the case with the multiple administration of a lower dose of STZ (in the mouse e.g., 40 mg/kg) (54). The severe side effects could be even worse in large animal models, such as non-human primates (55, 56).

Alloxan is an urea derivative that has diabetic effects by inducing necrosis of pancreatic β cells. It is mainly used in rabbits, rats, mice and dogs. In addition, by regulating the dosage one can also regulate the severity of the disease. Alloxan and its reduction form dialuric acid also contribute to the formation of superoxides, driving the formation of hydrogen peroxide. This makes the chemical a frontrunner in the study of the links between ROS and diabetes.

A lesser known chemical is ferric nitrilotracetate, which can induce hyperglycemia, ketonemia, and ketonuria after administration in rats and rabbits (51).

In this context, the administration of contra-insulin hormones should be mentioned as an induction model as well. Hormones such as epinephrine, glucagon, glucocorticoids, and growth hormones have an antagonistic effect on insulin. Significant elevation of, for example, epinephrine and glucagon can induce hyperglycemia and ß-cell hyperplasia.

Surgical approaches, such as removal of pancreatic cells, have also found their application in the past, e.g., in rats.

#### 2.1.3. Genetic Rodent Models

First, the Zucker diabetic fatty rats (ZDF rats), developed by a mutation in the leptin receptor gene, have to be mentioned. Not only the diabetically important changes in the endocrine and metabolic balance characterize this rat line, but also variations in behavior and neuronal changes. They are primarily used in cardiovascular research because they express hypertension, sympathetic disturbances and fibrosis on cardiac activity in addition to diabetes (57).

Genetic models in mice are manifold as they are easy to establish and also remain manageable due to the short gestation period. Among these would be the OVE26 mouse as a model for T1DM and the *ob/ob* as well as *db/db* mouse lines for T2DM (25). OVE26 mice are characterized by an overexpression of the Ca^2+^-binding protein calmodulin in pancreatic β-cells, which in turn causes sustained damage to these cells. The obese mouse line *ob/ob*, or *Lep*^*ob*^, is characterized by a monogenetic inheritance mutation on chromosome 6 in C57BL/6J. Like ZDF rats, the mutation affects the gene encoding leptin, causing homozygous mice of this strain to extensively gain weight and suffer from insulin intolerance over time. When this mutation occurs in the C57BL/KS line, diabetes is much more aggressive, resulting in regression of islets and premature death of the animals (26). The *db/db* mouse, or *Lepr*^*db*^, goes back to the same background. However, these mice develop hyperglycemia from 4 to 8 weeks of age due to β-cell failure. *db/db* mice usually reach an age of only 8–10 months. In combination with other transgenic alterations (e.g., ApoE^−/−^ or LDLR^−/−^) by crossing with the corresponding C57BL/6 strains, these two mouse models are used in artherosclerotic research and other applications (58, 59). The Kuo Kondo (KK) mouse, as well as the corresponding rat line, is a polygenic line and can be used as a model for T2DM (60). This line is characterized primarily by obesity, hyperinsulinemia and hyperglycemia. Strains modified from this are predominantly used in the testing of new diabetes drugs.

### 2.2. Non-mammalian Models

The first animal model that comes to mind with this heading is the widely used zebrafish (*Danio rerio*). Zebrafish is an ideal laboratory model, because, in addition to the relatively uncomplicated husbandry conditions, they also offer some advantages in experimental performance. For example, the embryos and larvae are nearly transparent, allowing a wide variety of optical measurement techniques, such as light sheet microscopy and *in vivo* optical mapping [see e.g., Bassi et al. (61)]. In addition to primarily developmental biology studies, the zebrafish has also been established in the field of cardiovascular diseases. Due to the similarities in physiological parameters, such as heart rate and repolarization rate, and the genetic possibilities, a true playground for experimental modeling of heritable and molecular cardiovascular diseases opens up (62, 63). However, it should not go unnoticed that this is still a fish. From an anatomical point of view, this means that the zebrafish has only one circulatory system and therefore only a two-chambered heart. Furthermore, this animal model is ectotherm, which means that all physiological and especially electrophysiological measurements must be considered temperature-critical. Likewise, different variations and compositions of ion channels are found in zebrafish compared to humans, which is why caution must be exercised in the transferability of pharmacological studies.

In addition to cardiovascular approaches, diabetic models have been manifested in zebrafish. Diet-induced obesity (DIO) leads to significant changes in insulin production and blood glucose levels in zebrafish, making this DIO model useful for T2DM research (29). Additionally, of course, genetic models exist for T1DM, which can also be induced by surgical removal of pancreatic cells or by administration of chemical agents (e.g., STZ) (64, 65).

Another widely used laboratory model is the clawed frog (*Xenopus laevis*). Primarily used for developmental cardiac questions, this aquatic inhabitant is also used, among other things, for the study of genetic diabetes with attention to pancreatic development (66, 67). Research in Xenopus is focused on congenital heart disease, long QT syndrome, hypertension and atrial fibrillation, whereby genetic modifications of cardiac ion channels can be studied in particular in oocytes. Due to the fact that not only ion channels but also pancreatic development is genetically highly conserved between Xenopus and humans, congenital prancreas defects can also be studied, which represents T1DM related symptoms (68, 69).

Similar to Xenopus, the model organism fruit fly (*Drosophila melanogaster*) also offers highly conserved genetic properties. Here, too, changes in the ion channel structure (channelopathies) or cellular dysfunctions (cardiomyopathies) can be illuminated, as they occur, for example, in congenital heart disease (70). In analogy to humans, Drosophila also shows a regulatory loop of glucose and lipid metabolism by Drosophila insulin-like peptides, thus offering considerations with regard to diabetic alterations (71). To characterize diabetic effects, DC-like symptoms can be induced by a high-fat or high-sugar diet, which in turn would correspond to a T2DM (30). Despite the genetic investigations, Drosophila also offers the possibility to observe irregularities of cardiac beating in its tubular heart system, which in turn permits conclusions with respect to genetically linked arrhythmia onset (71).

Despite all the genetic advantages, the short experimental periods due to short lifetimes as well as the handling-specific advantages, it should be noted that the construction of the mammalian heart differs from that of the non-mammalian heart, which ultimately limits the morphology and cardiac electrophysiology. However, zebrafish, Xenopus, and Drosophila offer useful and exciting insights into the primarily genetic cardiovascular and diabetic relationships.

At this point it should not go unmentioned that there are other aquatic living animal models besides the zebrafish and the clawed frog. For example, Venn-Watson et al. (72) also describe the use of dolphins as a diabetic model. However, such models seem to be highly unlikely to be feasible in all diabetic working laboratories.

### 2.3. Large Animal Models

Large animal models are well known for many translational pre-clinical approaches because of their high anatomic and physiologic similarity to humans. Due to their long lifespan compared to small animals, they are also particularly suitable for long-term studies. Regarding diabetes research, in this group of laboratory animals is mainly found spontaneous, chemically induced and DIO based diabetes. Thus, diabetes mellitus is a common condition in dogs, defined by persistent hyperglycemia and insulin deficiency due to β-cell loss (73). Besides, cats also exhibit spontaneous T2DM (74). Both also show an increase in the number of cases in domestic pets, which is probably due to DIO. Large animal clinical models in cardiology have been particularly prominent in farm animals, such as pigs (*Sus scrofa*), sheep (*Ovis spec*.), and cattle (*Bos taurus*) [see e.g., (75–78)]. Also, clinical trials are now often flanked by experiments in non-human primates. In the combination of diabetes and cardiovascular disease, the porcine and NHP models have prevailed.

#### 2.3.1. Porcine Models

As a clinically relevant large animal model, pigs are very well integrated into everyday research. Thus, methods/techniques can be applied to them which are already used in human medicine without having to adapt them to the size of the animal. Furthermore, pigs are well known for their extensive weight gain and also for obesity. Although, spontaneously occurring cases of diabetes are rather rare in pigs (79). To establish a suitable diabetes model in pigs one has to resort to chemical induction, surgical methods or genetic engineering. Once induced, however, porcine models offer a tremendous variety of cardiac interventions. Additionally, the use of minipigs also offers the possibility to observe diabetic changes in the cardiovascular system on a long time scale. Well-known diabetic and hypercholesteralemic pig models, respectively, are the DM model, the LDLR^−/−^ model for familial hypercholesterolemia (Rapacz-Pig), the gain-of-function mutant pig (PCSK9) as well as the metabolic disease model (Ossabaw Pig) (80). These models are induced on the one hand by DIO based methods and on the other hand by genetic modifications in Minipig lines. Besides, there are also tailored genetically modified procine models for specific simulation of human diseases, such as diabetes mellitus or chronic inflammation patterns like artherosclerosis (81). Here one founds manifold possibilities of genetic modifications to investigate the direct effect of diabetic conditions on the cardiovascular system. Genetic models range from modification of specific receptors (e.g., dominant-negative glucose-dependent insulinotropic polypeptide receptor—GIPR^*dn*^) (82) to influencing insulin expression (83, 84) to β-cell specific expressions (35). The further possibility of multitransgenic approaches makes genetic diabetes models in pigs even more attractive for cardiovascular research.

Moreover, there are the chemically induced diabetes models in the domestic pig. Here, mainly the STZ based method is applied (19). In these “chemical” models, especially the influence of oxidative stress and inflammation can be investigated. As for the mice models there are several protocols in respect to the different dosages of STZ, but since the by STZ affected glucose transporter 2 shows a rather low expression in pigs compared to rodents this approach can become a quite difficult venture (35). Furthermore, the porcine models can also be used for the testing of new genetic therapy approaches. Last but not least, there are the surgical methods; meaning the complete removal of the pancreas (pancreatectomy), which are rather used as models for β-cell transplantation (85, 86). While this simulates a complete loss of β-cells, it also loses the endocrine effect of the pancreas (87). Not to mention the surgical consequences in terms of recovery period and analgesia.

#### 2.3.2. Non-human Primates

Monkeys as experimental animal models are usually still rather the exception, yet they in particular show a high similarity to the human disease type in terms of physiological and also immunological characteristics (88, 89). Due to this, they are also common in xenotransplantation projects along with the previously mentioned pigs. Non-human primates (NHP) are a very good example of spontaneously occurring diabetes (31, 90). In this regard, macaques in particular have been studied in detail. Spontaneous diabetes has been detected in cynomolgus macaques (*Macaca fascicularis*), rhesus macaques (*Macaca mulatta*), black Celebes macaques (*Macaca nigra*), bonnet macaques (*Macaca radiata*), Formosan rock macaques (*Macaca cyclopis*) and pig-tailed macaques (*Macaca nemestrina*) (32). However, mainly the cynomolgus and rhesus macaques have become the object of study (91). Approximately 30 % of cynomolgus macaques develop hyperinsulinemia and glucose tolerance in old age (from 15 years on), and the monkeys with transition from glucose intolerance to T2DM are usually obese. Moreover, cynomolgus macaques are more likely to be nonketotic. Other features include an increase in blood pressure and inflammation rates, as well as blood cholesterol levels. The situation is similar in the rhesus monkeys. There, T2DM presents as a progressive disorder, with the monkeys showing a decrease in insulin sensitivity, response, and energy expenditure with increasing age. In addition, cases of gestational diabetes have also been demonstrated in both macaques.

Baboons are a very common model in cardiovascular research, but they are also prone to obesity and diabetes (T2DM). Furthermore, in baboons, glycemic and weight information is also heritable, making them a very good model for genotyping research.

To develop a NHP model, especially DIO with high amounts of fructose or transfatty acids (cynomolgus and rhesus macaques), as well as sucrose and fat (baboons) can be applied. NHP models very often develop atherosclerosis and the cardiovascular diseases favored by it. In addition to the nutritionally induced types, there is also evidence for the effect of psychosocial stress on the development of obesity and diabetes in NHP (92).

Despite these characteristics, NHPs also represent a very specific animal model. So, the ethical and legal requirements for research projects involving monkeys are much higher than for mouse experiments. In addition, the specific housing conditions and the associated specification of personnel are not feasible at every animal facility. Nevertheless, they represent a highly translatable animal model for which participation is well worth the effort to overcome the hurdles.

## 3. Choose Wisely–Basic vs. Translational?

**Figure 1:**
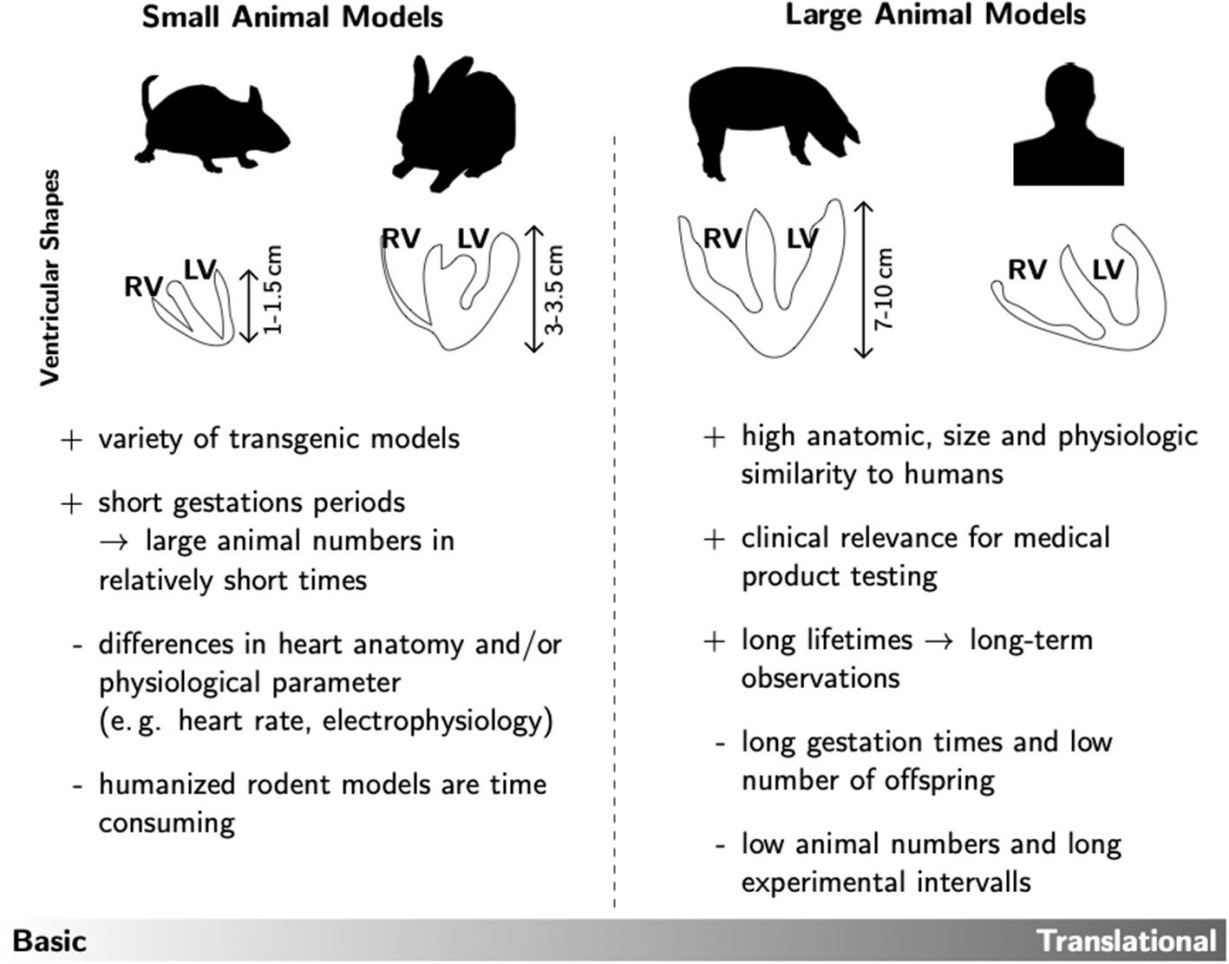
Overview of the main advantages (+) and disadvantages (–) of small and large animal models. As prominent examples for each group the ventricular shapes of mice, rabbits, pigs and humans are shown. This rather technical drawing illustrates that the different species not only differ in heart size, but also in ventricular wall geometry. Such anatomic specifications could have significant influence on the experimental testing of prospective therapeutic approaches in e.g., cardiac rhythm treatment.

As already mentioned, the question of the right animal model always depends on the actual research question. Each model has its advantages and disadvantages, so that one must inevitably formulate the hypothesis of the investigation first. For example, fundamental mechanisms and other relationships in basic research can be represented very well in small animal models. The advantages here are obvious, since there are a large number of genetically modified models available, especially in mouse lines, and the gestation times are also manageable, making replicative series of experiments much easier. Investigation methods are also very well established and widely used in this group of laboratory animals. In addition, the handling of small animals is easily feasible in most laboratories. However, small laboratory animals in particular also harbor pitfalls. For example, the ventricular shapes differ significantly from the human ideal, not to mention the size (see Figure 1). This can play an important role if the focus is not only on diabetic changes, but also on cardiac remodeling and the resulting arrhythmia development. Here, the size of the heart as well as the ventricular wall thickness is a crucial factor for arrhythmic excitation propagation, because due to the spatiotemporal propagation patterns of vortex-like tachycardiac excitations, the degree of pronounced complexity and the associated termination method or, respectively, probability is limited. Moreover, some representatives of small laboratory animals show differences in the composition of their cardiac ion channels as well as differences in electrophysiology (e.g., heart rate, action potential duration) [see e.g., (93, 94)]. Nevertheless, transgenic and pharmacological small animal models are well suited for the study of underlying cellular and molecular mechanisms, as well as for the identification of targets for translational (pre-) clinical research.

In pre-clinical research, large animal models find their main application, due to their high anatomical and physiological similarity to the human cardiovascular system. For example, pigs exhibit vascular anatomy and blood flow through the coronary vessels comparable to 90 % of the population, and collaterization is also similar to that of humans (95). Furthermore, primates show e.g., a high similarity in the immune system, which makes them especially suitable for testing novel gene therapies or even xenotherapies (96).

However, there are obvious disadvantages here as well. For example, large animal models are often characterized by long gestation periods and a low number of offspring, making these experiments marked by both low animal numbers and long experimental times. Also, the handling of large animals, especially primates, cannot be established everywhere and often requires extensive specialization. But considering the clinical relevance with regard to the testing of new therapeutic approaches for human medicine, especially the large animal models are indispensable, since they can score not only by their anatomical similarities but also by the use of surgical techniques already established in human medicine. This not only facilitates the translation of successfully tested approaches later on, but also their acceptance by the cardiological community and the patient.

## 4. Conclusion

In summary, only one thing can be said about the choice of the most suitable animal model: There is no such thing like an ideal model for the illustration of human disease patterns. Rather, one should pay attention to the intention of one's research project. Questions about reproducibility and applicable methods, safe handling of the species according to the guidelines of the 3R (in means of reduction, refinement and replacement), specialization in personnel and technology should be given equal importance to scientific questions. Especially with regard to animal welfare and the guidelines of the 3R, it should be clarified in advance which model promises the best results for the research question. It does no one any good if pre-clinical translational approaches are tested to exhaustion in rodent models and then fail miserably in clinical trials due to physiological differences not considered beforehand (87, 97). Taking into account the considerations mentioned in section 3, which mainly include the property of therapeutic translation, the following critical considerations can be made for the establishment and investigation of diabetic and cardiovascular animal models. I. What exactly do I want to study? Also this seems obvious, this actually reflects an important point. Molecular processes and the influence of cellular pathways can be investigated very well in small animal models, since a wide range of transgenic models is already available for these and, due to the short gestation periods, statistical validations can also be carried out at predictable intervals. In contrast, long-term studies with experimental durations of months or even years are hardly possible in small animal models. In addition, the application of instruments from human medicine (e.g., cardiac catheters or implants) is not easily possible without prior adaptation. Especially for the heart it should be mentioned again that different species can have different anatomical and electrophysiological parameters. II. Which burden of the animal is opposed to which gain of knowledge? The assessment of the expected or actual burden on the animals is probably one of the most difficult considerations. In addition to possible genetically or protocol induced stress (pain, suffering, fear, etc.), factors such as experimental duration and thus the overall study design are also taken into account. Likewise, this consideration is also the time for questioning repetition, i.e., does this experiment represent a repetition of a previous experiment, possibly conducted by another group or are not yet investigated research questions answered. In general, it can be said that the reduction of the load and the number of animals are the most important. This means that if it is possible to achieve the objective of the study by less invasive methods, these should be used.

Nevertheless, in our view, the use of experimental animals and the simulation of diseases such as diabetes and cardiomyopathy are invaluable. Therefore, we want to sum up with the words of E. Shafrir extending them to the field of cardiovascular research as well.

“There is no need to underscore the enormous contribution of diabetes research in animals to the understanding of various aspects of human diabetes. Although none of the animals fully represents the human syndrome, the various mechanisms and complications in animals are worth testing because human diabetes also shows many variations.” [Preface of (98)].

## Author Contributions

CR conceptualized, wrote, and edited the manuscript. RH reviewed and edited the manuscript. Both authors agree to be accountable for the content of the work.

## Conflict of Interest

The authors declare that the research was conducted in the absence of any commercial or financial relationships that could be construed as a potential conflict of interest.

## Publisher's Note

All claims expressed in this article are solely those of the authors and do not necessarily represent those of their affiliated organizations, or those of the publisher, the editors and the reviewers. Any product that may be evaluated in this article, or claim that may be made by its manufacturer, is not guaranteed or endorsed by the publisher.

## Abbreviations

AF: atrial fibrillation
DC: diabetic cardiomyopathy
DIO: diet-induced obesity
NHP: Non-Human Primates
T1DM: type 1 diabetes mellitus
T2DM: type 2 diabetes mellitus
ROS: reactive oxygen species
STZ: streptozotocin
VF: ventricular fibrillation
ZDF: Zucker diabetic fatty.
